# Sensory and Instrumental Flavor Changes in Green Tea Brewed Multiple Times

**DOI:** 10.3390/foods2040554

**Published:** 2013-11-29

**Authors:** Jeehyun Lee, Delores Chambers, Edgar Chambers

**Affiliations:** 1 Department of Food Science and Nutrition, Pusan National University, 30 Jangjeon-Dong, Geumjeong-gu, Busan 609 735, Korea; E-Mail: jeehyunlee@pusan.ac.kr; 2 Sensory Analysis Center, Kansas State University, Manhattan, KS 66506-1407, USA; E-Mail: eciv@ksu.edu

**Keywords:** green tea, multiple brews, flavor, sensory, volatile compounds

## Abstract

Green teas in leaf form are brewed multiple times, a common selling point. However, the flavor changes, both sensory and volatile compounds, of green teas that have been brewed multiple times are unknown. The objectives of this study were to determine how the aroma and flavor of green teas change as they are brewed multiple times, to determine if a relationship exists between green tea flavors and green tea volatile compounds, and to suggest the number of times that green tea leaves can be brewed. The first and second brews of the green tea samples provided similar flavor intensities. The third and fourth brews provided milder flavors and lower bitterness and astringency when measured using descriptive sensory analysis. In the brewed liquor of green tea mostly linalool, nonanal, geraniol, jasmone, and β-ionone volatile compounds were present at low levels (using gas chromatography-mass spectrometry). The geraniol, linalool, and linalool oxide compounds in green tea may contribute to the floral/perfumy flavor. Green teas in leaf form may be brewed up to four times: the first two brews providing stronger flavor, bitterness, and astringency whereas the third and fourth brews will provide milder flavor, bitterness, and astringency.

## 1. Introduction

The flavor, aroma, and appearance are the basic sensory components of green tea [1]. Green tea is consumed by many people living in Asia for its flavor, cultural connotations, and health benefits. Recently, western countries are embracing green tea as a beverage because of the possible health benefits [2]. In addition to the health effects of green tea, researchers have studied the flavors of green tea using descriptive sensory methods [3,4,5,6]. Recently, a green tea lexicon was developed evaluating over 100 green tea samples [7]. The aromatic volatile composition of green teas also has been studied extensively by researchers [8,9,10,11,12,13,14,15].

While the results of this previous research have been valuable, the researchers evaluated only the first brews of green tea. It is generally believed in Asia that a high quality green tea can be brewed multiple times. A Chinese poet sang about tea drinking and mentioned the first through the seventh brew [1]. Many premium and high end products suggest brewing their tea multiple times (up to five times) in the infusion directions. Kim [16] suggested that green tea can be brewed up to three times. However, some of the literature on the multiple brewing of green tea is contradictory. Several researchers recommended using fresh leaves or a new tea bag when wanting more tea instead of using already brewed tea leaves or a tea bag and stated that used leaves do not provide much more than a little bitterness [17]. This may be true for green tea bags because such products are processed specifically to increase the surface area to extract the flavor and nutrients quickly. But no such study has been made on the similar properties of green tea that is brewed from tea leaves.

A limited number of researchers have reported on teas that have been brewed more than once [18,19]. The concentrations of caffeine, theobromine, and theophylline were measured in tea samples brewed three times [18]. The percentage of caffeine released in each brew of tea decreased as they were brewed repeatedly. However, the flavor and aroma changes of green tea as it is brewed repetitively were not discussed in the research literature. Recent research showed that brewing methods impact green tea flavor, but those authors did not test multiple brewing [20].

Investigating the aroma and flavor characteristics of green teas liquors that have been brewed repetitively can help determine how many times green teas can be brewed for consumption. No literature was found indicating how the aromatic volatile compounds and the flavor characteristics change when green tea is brewed multiple times. Thus, the objectives of this study were to: (1) determine how the flavor changes as green tea is brewed multiple times, (2) determine how the aromatic volatile compounds change as green tea is brewed multiple times, (3) relate the descriptive flavor analysis of green tea flavors to the aromatic volatile compounds data at each brew, and (4) suggest the number of times that green tea leaves can be brewed and still maintain their major flavor notes.

## 2. Experimental

### 2.1. Descriptive Sensory Evaluation

#### 2.1.1. Tea Samples

Six green tea samples were obtained from four different Korean green tea manufacturers (Table 1). These were selected based on availability, package directions with various brews recommended, and tea quality. The tea quality was determined based on the retail price, the higher prices reflecting higher tea qualities [15]. All six samples are considered either premium or high quality.

Ten grams of each green tea sample were placed in a warmed porcelain tea pot. Then 300 mL of 70 °C ultra pure water was added to the pot and the tea was brewed for 2 min and, while it brewed, the pot was swirled 10 times. The tea was poured through a porcelain strainer and served in white porcelain tea cups. The International Organization for Standardization [21] suggested the white porcelain tea wares to maintain a consistent result. After the first brew the tea leaves remained in the pot. Brewing was repeated until a total of five brews from the same tea leaves had been tested.

**Table 1:** Product name, manufacturer, harvest date, and price for green tea samples evaluated.

Green Tea Samples	Manufacturer	Harvest Date	Retail Price		Recommended Number of Brews
			Korean Won	US Dollar	
*Daehan Ujeon*	Daehan Tea	~April 20	70,000/100 g	69.14/100 g	Up to 3 times
*Daehan Ujeon Wild*	Daehan Tea	~April 20	Not available for retail sale	Not available	Not available
*Illohyang*	Amorepacific, Co.	~April 5	100,000/60 g	164.62/100 g	Multiple times to taste
*Myoungjeon*	Myoungsul Cha	~April 20	150,000/80 g	185.19/100 g	4–5 times
*Ouksu*	Amorepacific, Co.	May 26	20,900/80 g	25.80/100 g	2–3 times
*Ujeon Okro*	Hwagae Jeda	April 16	50,000/40 g	123.46/100 g	Not available

#### 2.1.2. Tea Preparation and Serving Procedure

Because of the nature of multiple brews, the serving order could not be randomized. Each tea sample was randomly assigned to a day and the five brews of the same tea were served in order. Between tea samples, unsalted-top crackers (Unsalted tops premium saltine crackers, Nabisco, East Hanover, NJ, USA) and reverse osmosis, deionized, carbon-filtered water were used to cleanse the palate. Three replications were completed on all the samples. 

#### 2.1.3. Descriptive Sensory Panel Evaluation

Six highly trained panelists from the Sensory Analysis Center at Kansas State University served as the panel. The panelists had completed 120 h of general training and had a minimum of 1200 h of general sensory testing including beverages, vegetables, and green tea. In addition, the panel had spent a total of 60 h developing a green tea lexicon with reference materials using a wide range of green teas [20] and had participated in testing more than 150 different samples of green tea. The panelists used a 0–15 point scale with 0.5 increments to evaluate the sensory attributes of the samples. Samples were evaluated in triplicate. Similar panels and testing procedures have been used recently for other products [22,23,24,25].

### 2.2. Gas Chromatography/Mass Spectrometry

#### 2.2.1. Tea Samples

The green tea samples were prepared in the same manner that they were prepared for the descriptive sensory analysis panel except for the type of water. Distilled water (Cincinnati, OH, USA) was used.

#### 2.2.2. Extraction of Volatile Compounds by SPME

For the extraction of the volatile compounds 10 mL of each green tea sample was transferred into a 40 mL amber headspace vial (Supelco; Bellefonte, PA, USA). Authors used 1,3-dichlorobenzene (Sigma-Aldrich, Milwaukee, WI, USA) as an internal standard for the quantification of the volatile compounds from the samples. One microliter of the internal standard (concentration: 200 ppm) was added. An octagonal, magnetic stir bar (diameter 8 mm × length 13 mm; Fisher; Pittsburgh, PA, USA) and analytical grade sodium chloride (*ca.* 3 g; Sigma-Aldrich) were added to the vial to help with the extraction. An open-center screw cap with a silicone/PTFE septum (22 mm diameter × 3.2 mm thickness, Supelco) was used to close the amber vial. Each sample was allowed to equilibrate at 60 °C in a Reacti-Therm™ heating block (Pierce Biotechnology, Inc.; Rockford, IL, USA) for 5 min. The volatile odor compounds from the sample were extracted using a StableFlex 50/30 µm three phase (DVB/CAR/PDMS) SPME fiber (Supelco) at 60 °C for 20 min.

#### 2.2.3. Gas Chromatography/Mass Spectrometry (GC-MS)

After extraction, the SPME fiber was retracted and the holder was moved to the splitless injection port of a 5890 Series II Gas Chromatograph (Hewlett-Packard Co.; Palo Alto, CA, USA) for manual injection. The fiber was desorbed for 5 min and the injection port was maintained at 225 °C. The volatiles were separated on an Rtx^®^-5 (Crossbond^®^ 5% diphenyl-95% dimethyl polysiloxane; 30 m length × 0.25 mm internal diameter × 0.25 μm film thickness) capillary column (Restek; Bellefonte, PA, USA). The temperature program for the separation was as follows: 40 °C for 1 min, 13 °C/min of ramp rate to 250 °C and held for 1 min. The total time was 18.15 min. The identification of the compounds was done using an HP 5890 Series II GC/HP 5972 mass selective detector (MSD, Hewlett-Packard Co.; Palo Alto, CA, USA) with the following parameters: interface temperature, 250 °C; ionization energy, 70 eV; mass range, 33–350 a.m.u.; scan rate, 2.2 scans/s. Ultra high purity Helium (AirGas; Westpoint, MS, USA) was used as a carrier gas at a constant flow rate of 0.96 mL/min. The mass spectra of the volatile compounds were compared using the Wiley138K Mass Spectral Database (Version B00.00, 1990; John Wiley and Sons, Inc.; New York, NY, USA). The volatile compounds were analyzed in triplicate for each sample. The Kovats retention indices (RI) were calculated to confirm the identification of the compounds. The concentrations for the green tea volatile compounds were calculated and reported on the basis of the internal standard concentration. 

### 2.3. Data Analyses

The descriptive data were analyzed using the repeated measures analysis of variance (ANOVA) to compare the subsequent brews to the first brew within each tea sample (SAS^®^ Version 9.1; SAS Institute; Cary, NC, USA). The mean scores of the volatile compounds concentration were calculated. To understand the flavor changes during multiple brews, the principal component analysis was conducted with the covariance matrix using PROC PRINCOMP statement in the SAS program. 

The mean scores of the descriptive analysis and the instrumental GC-MS evaluation at each brew were analyzed using the partial least square regression (PLSR2; Unscrambler^®^ 9.7, CAMO Software Inc.; Woodbridge, NJ, USA.) to determine the relationship between the descriptive data and the instrumental data. The covariance matrix was used in the PLSR analysis. 

## 3. Results and Discussion

### 3.1. Descriptive Sensory Analysis

Twenty-five of the attributes previously reported for green tea [7] were detected and evaluated for the six samples and five brews tested in this study. It is important to view these teas in terms of the description of those attributes as that can help in understanding the sensory profile of the teas. Those attributes were green, asparagus, celery, green herb-like, parsley, spinach, brown, ashy/sooty, burnt/scorched, tobacco, citrus, floral/perfumy, fruity, grain, medicinal, musty/new leather, nutty, seaweed, straw-like, sweet aromatics, bitter, astringent, and tooth-etch. Some attributes were similar to those found in three other Korean green teas [26] which were bitter, floral, cut grass, roasted grain, dried straw, burnt leaf, and astringency based on the term and the definition. The mean scores for all 25 attributes are shown in Table 2.

Green, brown, bitter, and astringent attributes were perceived throughout all five brewings of the green tea samples with a few exceptions at the fifth brew. Spinach, straw-like, and toothetch usually were present in at least the first three brews (Table 2). The intensity of the green flavor generally decreased as the samples were brewed repeatedly. The change in the intensity of spinach flavor was similar to the pattern of the green flavor. In general, the brown flavor intensity decreased from the second brew onward. The intensity of brown note in the first brew and the second brew were not statistically different. The intensity of straw-like decreased as green tea was brewed repetitively, except in the *Myoungjeon* sample where the highest intensity was in the second brew. The bitterness increased significantly from the first brew to the second brew in the *Daehan Ujeon*, *Daehan Ujeon Wild*, *Myoungjeon*, and *Ouksu* samples. The *Illohyang* and *Ujeon Okro* samples had similar bitterness intensities in both the first and the second brews. Beyond the second brew, the bitterness decreased significantly with each brew for all the samples. This is in partial agreement with findings that the percentage of caffeine, which is the major bitter substance in green tea, found in the green tea liquor decreased as the tea was brewed three times repeatedly [18]. The astringency and toothetch had similar patterns to the bitterness with the intensity being the highest at the second brew and then subtly decreasing with each subsequent brewing. 

**Table 2:** Mean scores and significant differences in flavor attributes for green tea brewed five times.

	*Daehan Ujeon*					*Daehan Ujeon Wild*					*Illohyang*				
	Brews					Brews					Brews				
	1	2	3	4	5	1	2	3	4	5	1	2	3	4	5
Green	4.03 ^a1^	3.31 ^b^	1.81 ^c^	0.91 ^d^	0.00 ^e^	2.67 ^a^	1.64 ^b^	1.44 ^b^	0.83 ^c^	0.64 ^c^	2.17 ^a^	1.42 ^b^	1.50 ^b^	0.94 ^c^	0.53 ^d^
Asparagus	1.66 ^a^	0.00 ^b^	0.00 ^b^	0.00 ^b^	0.00 ^b^	0.00	0.00	0.00	0.00	0.00	0.00	0.00	0.00	0.00	0.00
Celery	0.00	0.00	0.00	0.00	0.00	0.00	0.00	0.00	0.00	0.00	0.00	0.00	0.00	0.00	0.00
Green beans	0.75 ^a^	0.78 ^a^	0.00 ^b^	0.00 ^b^	0.00 ^b^	1.36 ^a^	0.00 ^b^	0.00 ^b^	0.00 ^b^	0.00 ^b^	0.00	0.00	0.00	0.00	0.00
Green herb-like	0.00	0.00	0.00	0.00	0.00	0.00	0.00	0.00	0.00	0.00	0.00	0.00	0.00	0.00	0.00
Parsley	0.00 ^b^	0.63 ^a^	0.00 ^b^	0.00 ^b^	0.00 ^b^	0.00	0.00	0.00	0.00	0.00	0.00	0.00	0.00	0.00	0.00
Spinach	1.88 ^a^	1.34 ^b^	0.69 ^c^	0.00 ^d^	0.00 ^d^	1.58 ^a^	1.22 ^ab^	0.82 ^b^	0.00 ^c^	0.00 ^c^	1.39 ^a^	0.72 ^b^	0.00 ^c^	0.00 ^c^	0.00 ^c^
Brown	2.75 ^a^	2.66 ^ab^	2.06 ^b^	1.13 ^c^	0.67 ^c^	3.83 ^a^	3.58 ^a^	2.03 ^b^	1.17 ^c^	0.53 ^d^	3.33 ^a^	3.47 ^a^	1.69 ^b^	1.00 ^b^	0.69 ^c^
Ashy/sooty	0.00 ^b^	0.50 ^a^	0.00 ^b^	0.00 ^b^	0.00 ^b^	0.00 ^c^	0.00 ^c^	0.94 ^a^	0.50 ^b^	0.00 ^c^	0.53 ^a^	0.86 ^a^	0.50 ^a^	0.00 ^b^	0.00 ^b^
Burnt/scorched	0.81 ^a^	1.09 ^a^	0.00 ^b^	0.00 ^b^	0.00 ^b^	1.11 ^a^	1.42 ^a^	0.00 ^b^	0.00 ^b^	0.00 ^b^	1.31 ^a^	1.22 ^a^	0.00 ^b^	0.00 ^b^	0.00 ^b^
Straw-like	1.41 ^a^	1.59 ^a^	1.25 ^a^	0.81 ^b^	0.00 ^c^	1.86 ^a^	1.89 ^a^	1.28 ^b^	0.72 ^c^	0.00 ^d^	1.44 ^a^	1.08 ^b^	0.92 ^b^	0.00 ^c^	0.00 ^c^
Tobacco	0.00 ^b^	0.00 ^b^	0.63 ^a^	0.00 ^b^	0.00 ^b^	1.00 ^ab^	1.36 ^a^	0.72 ^b^	0.00 ^c^	0.00 ^c^	1.19 ^a^	0.56 ^b^	0.00 ^c^	0.00 ^c^	0.00 ^c^
Citrus	0.00	0.00	0.00	0.00	0.00	0.00	0.00	0.00	0.00	0.00	0.00 ^b^	0.00 ^b^	0.75 ^a^	0.00 ^b^	0.00 ^b^
Floral/perfumy	0.00 ^b^	0.69 ^a^	0.84 ^a^	0.63 ^a^	0.56 ^a^	0.00	0.00	0.00	0.00	0.00	0.83 ^bc^	1.19 ^a^	1.17 ^ab^	0.83 ^bc^	0.56 ^c^
Fruity	0.72 ^a^	0.00 ^b^	0.00 ^b^	0.00 ^b^	0.00 ^b^	0.00	0.00	0.00	0.00	0.00	0.00	0.00	0.00	0.00	0.00
Grain	0.00	0.00	0.00	0.00	0.00	0.00	0.00	0.00	0.00	0.00	0.00 ^b^	0.64 ^a^	0.00 ^b^	0.00 ^b^	0.00 ^b^
Medicinal	0.00	0.00	0.00	0.00	0.00	0.00	0.00	0.00	0.00	0.00	0.00	0.00	0.00	0.00	0.00
Musty/new leather	0.00	0.00	0.00	0.00	0.00	0.00	0.00	0.00	0.00	0.00	0.00	0.00	0.00	0.00	0.00
Nutty	0.00 ^b^	0.66 ^a^	0.00 ^b^	0.00 ^b^	0.00 ^b^	0.00	0.00	0.00	0.00	0.00	0.00	0.00	0.00	0.00	0.00
Seaweed	2.28 ^a^	0.56 ^b^	0.00	0.00	0.00	2.28 ^a^	0.86 ^b^	0.00 ^c^	0.00 ^c^	0.00 ^c^	2.28 ^a^	0.00 ^b^	0.00 ^b^	0.00 ^b^	0.00 ^b^
Sweet aromatics	0.57 ^a^	0.00 ^b^	0.00 ^b^	0.00 ^b^	0.00 ^b^	0.00	0.00	0.00	0.00	0.00	0.53 ^a^	0.50 ^a^	0.00 ^b^	0.00 ^b^	0.00 ^b^
Bitter	6.91 ^b^	7.38 ^a^	5.63 ^c^	3.69 ^d^	2.34 ^e^	6.14 ^b^	6.72 ^a^	5.22 ^c^	3.64 ^d^	2.33 ^e^	6.44 ^a^	6.72 ^a^	5.47 ^b^	3.60 ^c^	2.47 ^d^
Astringent	1.81 ^a^	2.22 ^a^	2.06 ^a^	1.28 ^b^	0.53 ^c^	1.81 ^ab^	2.14 ^a^	1.69 ^ab^	1.53 ^b^	0.72 ^c^	1.58 ^b^	2.06 ^a^	2.03 ^a^	1.31 ^b^	1.00 ^c^
Tooth-etch	0.88 ^a^	1.31 ^a^	1.06 ^a^	0.59 ^b^	0.00 ^c^	1.14 ^a^	1.17 ^a^	0.89 ^ab^	0.67 ^bc^	0.50 ^c^	0.61 ^b^	1.03 ^a^	0.89 ^ab^	0.53 ^b^	0.00 ^c^
	*Myoungjeon*					*Ujeon Okro*					*Ouksu*				
	**Brews**					**Brews**					**Brews**				
	**1**	**2**	**3**	**4**	**5**	**1**	**2**	**3**	**4**	**5**	**1**	**2**	**3**	**4**	**5**
Green	3.97 ^a^	2.36 ^b^	2.03 ^bc^	1.64 ^c^	1.11 ^d^	2.51 ^a^	1.17 ^b^	0.57 ^c^	0.00 ^d^	0.00 ^d^	4.21 ^a^	4.24 ^a^	2.77 ^b^	1.62 ^cd^	1.42 ^d^
Asparagus	0.00	0.00	0.00	0.00	0.00	0.59 ^a^	0.00 ^b^	0.00 ^b^	0.00 ^b^	0.00 ^b^	0.00	0.00	0.00	0.00	0.00
Celery	0.00	0.00	0.00	0.00	0.00	0.00	0.00	0.00	0.00	0.00	0.00 ^b^	0.00 ^b^	0.50 ^a^	0.00 ^b^	0.00 ^b^
Green beans	1.14 ^a^	0.00 ^b^	0.00 ^b^	0.00 ^b^	0.00 ^b^	0.00	0.94	0.00	0.00	0.00	0.00 ^b^	1.03 ^a^	0.97 ^a^	0.00 ^b^	0.00 ^b^
Green herb-like	0.00 ^b^	0.00 ^b^	0.00 ^b^	0.00 ^b^	0.64 ^a^	0.00	0.00	0.00	0.00	0.00	0.00	0.00	0.00	0.00	0.00
Parsley	0.00	0.00	0.00	0.00	0.00	0.00	0.00	0.00	0.00	0.00	0.00 ^b^	1.12 ^a^	0.00 ^b^	0.00 ^b^	0.00 ^b^
Spinach	2.08 ^a^	1.92 ^a^	1.28 ^b^	0.72 ^c^	0.00 ^c^	1.78	0.00	0.00	0.00	0.00	2.59 ^a^	2.53 ^a^	0.89 ^b^	0.79 ^b^	0.56 ^b^
Brown	3.97 ^a^	4.44 ^a^	2.36 ^b^	1.47 ^c^	1.11 ^c^	3.25 ^ab^	3.75 ^a^	3.03 ^ab^	2.59 ^c^	1.69 ^d^	2.28 ^ab^	2.64 ^a^	1.14 ^c^	1.55 ^bc^	0.00 ^d^
Ashy/sooty	0.00 ^c^	1.39 ^a^	1.00 ^ab^	0.67 ^b^	0.00 ^c^	0.00 ^d^	2.59 ^a^	2.63 ^a^	1.88 ^b^	1.28 ^c^	0.00 ^b^	0.00 ^b^	0.00 ^b^	0.59 ^a^	0.00 ^b^
Burnt/scorched	2.17 ^a^	2.33 ^a^	0.69 ^b^	0.00 ^c^	0.00 ^c^	1.63 ^a^	0.50 ^b^	0.00 ^c^	0.00 ^c^	0.00 ^c^	1.26 ^b^	2.35 ^a^	0.00 ^c^	0.00 ^c^	0.00 ^c^
Straw-like	1.17 ^bc^	2.00 ^a^	1.50 ^ab^	0.89 ^cd^	0.64 ^d^	1.90 ^a^	1.77 ^ab^	1.43 ^b^	1.43 ^b^	0.74 ^c^	1.52 ^a^	1.44 ^a^	0.94 ^b^	0.76 ^b^	0.00 ^c^
Tobacco	0.00	0.00	0.00	0.00	0.00	0.00 ^c^	1.06 ^a^	0.94 ^ab^	0.50 ^b^	0.00 ^c^	0.00 ^b^	0.00 ^b^	0.00 ^b^	0.65 ^a^	0.00 ^b^
Citrus	0.00	0.00	0.00	0.00	0.00	0.00	0.00	0.00	0.00	0.00	0.00	0.00	0.00	0.00	0.00
Floral/perfumy	0.00	0.00	0.00	0.00	0.00	0.00	0.00	0.00	0.00	0.00	0.00	0.00	0.00	0.00	0.00
Fruity	0.00	0.00	0.00	0.00	0.00	0.00	0.00	0.00	0.00	0.00	0.00	0.00	0.00	0.00	0.00
Grain	0.00	0.00	0.00	0.00	0.00	0.00	0.00	0.00	0.00	0.00	0.00	0.00	0.00	0.00	0.00
Medicinal	0.00 ^b^	0.89 ^a^	0.94 ^a^	0.53 ^a^	0.00 ^b^	0.00	0.00	0.00	0.00	0.00	0.00	0.00	0.00	0.00	0.00
Musty/new-leather	0.00 ^c^	1.58 ^a^	0.72 ^b^	0.53 ^b^	0.00 ^c^	0.00	0.00	0.00	0.00	0.00	0.00	0.00	0.00	0.00	0.00
Nutty	0.00 ^b^	0.56 ^a^	0.00 ^b^	0.00 ^b^	0.00 ^b^	0.00	0.00	0.00	0.00	0.00	0.00	0.00	0.00	0.00	0.00
Seaweed	4.19 ^a^	0.69 ^b^	0.00 ^c^	0.00 ^c^	0.61 ^b^	2.22 ^a^	0.00 ^b^	0.00 ^b^	0.00 ^b^	0.00 ^b^	1.91 ^a^	0.85 ^b^	0.00 ^c^	0.00 ^c^	0.00 ^c^
Sweet aromatics	0.00	0.00	0.00	0.00	0.00	0.00 ^b^	0.00 ^b^	0.56 ^a^	0.00 ^b^	0.00 ^b^	0.00 ^b^	0.00 ^b^	0.71 ^a^	0.00 ^b^	0.59 ^a^
Bitter	7.14 ^b^	8.56 ^a^	6.25 ^c^	4.75 ^d^	3.42 ^e^	6.08 ^ab^	6.39 ^a^	5.61 ^b^	4.48 ^c^	3.54 ^d^	6.99 ^b^	7.84 ^a^	5.46 ^c^	3.93 ^d^	2.69 ^e^
Astringent	1.78 ^b^	2.83 ^a^	2.00 ^b^	1.42 ^c^	0.92 ^d^	1.32 ^b^	1.79 ^a^	1.54 ^b^	0.75 ^c^	0.57 ^c^	1.64 ^b^	1.97 ^a^	1.38 ^b^	0.94 ^c^	0.00 ^d^
Tooth-etch	0.81 ^b^	1.78 ^a^	1.06 ^b^	0.83 ^b^	0.00 ^c^	0.53 ^a^	0.81 ^a^	0.66 ^a^	0.00 ^b^	0.00 ^b^	0.53 ^b^	0.81 ^a^	0.66 ^b^	0.00 ^c^	0.00 ^c^

a–e, Means within same row of each green tea sample with different superscripts are different (*p* < 0.05) when analyzed using repeated measures analysis of variance.

In addition to the green and spinach flavor notes, other green-related attributes were perceived: asparagus, celery, green beans, green herb-like, and parsley. The asparagus note was barely detected in the first brews of the *Daehan Ujeon* and *Ujeon Okro* samples. All the samples, except *Illohyang*,had green beans at barely detected to low levels in the first, the second, or the third brew. The celery was present in the third brew of the *Ouksu* sample. The fifth brew of the *Myoungjeon* sample had a green herb-like note at threshold level. The parsley was present in the *Daehan Ujeon* and *Ouksu* samples in the second brews. 

Besides the brown and straw-like attributes, the brown-related attributes that were perceived were ashy/sooty, burnt/scorched, and tobacco. An ashy/sooty note was perceived in all of the samples but mainly in the second, the third, and the fourth brews. The burnt/scorched flavor was perceived at barely detected to low levels, the intensity ranging 0.5 to 2.35, mainly in the first and the second brews of all the Korean green teas. The tobacco was perceived in all the samples other than *Myoungjeon*, mostly in the second and the third brews. 

Other attributes that were detected in the samples included citrus, floral/perfumy, fruity, grain, medicinal, musty/new leather, nutty, and seaweed. Many of these sensory attributes are present only in some teas and only during some brews, meaning that in many cases the attribute was not present and is shown by 0.0 in the table. This is not surprising given the unique nature of the falvor of green teas and the potential for the sensory properties to change during multiple brews of each tea.

The citrus was detected in the third brew of the *Illohyang* sample. A floral/perfumy note was detected only in two of the samples, *Daehan Ujeon* and *Illohyang*, and was present from the second brew onward for the *Daehan Ujeon* sample and in all the five brews for the *Illohyang* sample at low intensities. The *Daehan Ujeon* sample was the only one perceived as having a fruity note, and that was only in the first brew. Grain flavor was found only in the *Illohyang* sample in the second brew. The *Myoungjeon* sample was perceived to have medicinal note at a threshold level in the second, the third, and the fourth brews. The musty/new leather note was only detected in *Myoungjeon* from the second brew to the fourth brew at very low levels. The nutty flavor was present only in the second brews of the *Daehan Ujeon* and *Myoungjeon* Samples. All six green tea samples had low seaweed flavors in the first brews and the *Daehan Ujeon*, *Daehan Ujeon Wild*, *Myoungjeon*, and *Ouksu* samples were perceived to have seaweed flavor at a low level in the second brews. The seaweed was also present in the fifth brew of the *Myoungjeon* sample. The seaweed flavor was first researched and documented in 1977 [3] and was later defined and referenced [7] to describe the flavor of some green teas. However, it was not reported by researchers who have previously studied Korean green tea [26,27]. A sweet aromatics note was present at the threshold level in four of the samples (*Daehan Ujeon*, *Illohyang*, *Ujeon Okro* and *Ouksu*) at various brews. 

**Figure 1:**
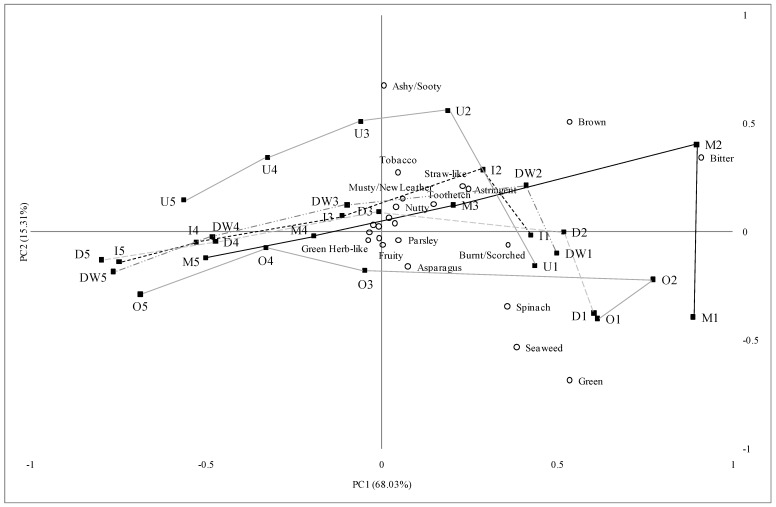
Principal component analysis biplot of descriptive sensory analysis of green teas ^a, b^.

^a^ D = *Daehan Ujeon*; DW = *Daehan Ujeon Wild*; I = *Illohyang*; M = *Myoungjeon*; O = *Ouksu*; U = *Ujeon Okro*; Numbers following the sample name denotes number of brew; ^b^ Lines connect consecutive brews of the same green tea samples to show the trend of flavor change.

The flavor of the green loose leaf tea samples changed when they were brewed multiple times. Generally, the flavor decreased as the number of brews increased in all the green tea samples in the current study. This trend is shown in PCA biplot (Figure 1). The first two brews appeared to provide similar intensities of the flavor attributes, except for the intensities of the green-related attributes which actually decreased and the intensities of bitterness, astringency, and toothetch which increased from the first to the second brews. 

While the numbers of flavor attributes and their intensities decreased beyond the second brew, the third brew and even the fourth brew may be enjoyed by consumers who prefer a milder flavor and low bitterness and astringency in their green tea. The fifth brew presented a few flavor notes at low levels and low bitterness and astringency, suggesting that most of the flavor is gone by the fifth brew. Also, as shown in Figure 1, the flavor changes occurring during the multiple brews of green tea is rather continuous and, because the flavors get weaker each time, the changes of flavor may appeal to some consumers. 

### 3.2. Aroma Volatile Compounds

Fourteen aroma volatile compounds were identified and quantified in the green tea samples brewed five times (Table 3). It must be remembered that these teas were extracted only with hot water and headspace was analyzed, much as tea might be smelled before tasting. Thus, because no chemical extraction was used and no intentional concentration of volatiles was done, the number of compounds is much lower than that reported in most previous studies. There were two aliphatic alcohols ((*z*)-4-hexen-1-ol and 2-ethyl-1-hexanol), two aromatic alcohols (benzenemethanol and benzeneethanol), two terpene alcohol (linalool and geraniol), one aliphatic aldehyde (nonanal), two aromatic aldehydes (benzaldehyde and phenylacetaldehyde), three ketones (4-methyl-3-penten-2-one, jasmone, and β-ionone), one furan (linalool oxide), and two other compounds (1H-indole and 1-ethyl-1H-pyrrole-2-carboxaldehyde).

**Table 3:** Fourteen volatile compounds found in six Korean green teas and Kovats retention indices calculation for the compounds.

Compounds	Kovats RI Calculated	Kovats RI Reported
4-Methyl-3-penten-2-one	815.8	800 [28]
(*z*)-4-Hexen-1-ol	873.3	868 ^a^
Benzaldehyde	985.3	962 [29]
2-Ethyl-1-hexanol	1047.6	1028 [30]
Benzenemethanol	1058.3	1033 [31]
Benzeneacetaldehyde	1058.3	1045 [30]
Linalool oxide	1094.8	1070 [32]
Linalool	1126.5	1100 [33]
Nonanal	1122.5	1103 [28]
Benzeneethanol	1140.5	1113 [34]
Geraniol	1234.0	1259 [35]
1H-Indole	1328.3	1286 [36]
Jasmone	1421.6	1394 [36]
β-Ionone	1405.4	1482 [30]

^a^ Estimated value in NIST library.

The concentrations of these compounds are shown in Table 4. The concentrations of the volatile compounds in the current study are generally lower than the threshold reported in previous literature.

We hypothesize that the reason for this is that green tea generally is consumed at a higher temperature whereas the typical threshold evaluation by previous researchers is conducted at room temperature. Also, the volatile compounds in tea are present in a complex matrix of volatiles whereas reported thresholds are for the simple compounds. The Kovats RI’s calculated based on retention time and previous reports by other researchers are shown in Table 3.

The compounds also do not automatically decrease or increase over time. Although some of this may be sampling error, it also is quite likely that changes in the leaves as the teas are repeatedly doused with hot water in subsequent brews are likely to affect the release of compounds. For example, nonanal appears to generally increase in most teas suggesting that the leaves are releasing more of the compound as the cells in the leaves are disrupted during subsequent brewing. However, in some teas the nonanal decreases in some later samples suggesting that for those teas most of the nonanal has been released in prior brews.

**Table 4:** Composition and concentration of the volatile compounds in six green teas infusion (μg/L).

Compounds	Brew 1	Brew 2	Brew 3	Brew 4	Brew 5
*Daehan Ujeon*					
Benzenemethanol	4.84	-	-	-	-
Linalool	-	3.48	12.97	17.29	-
Nonanal	7.35	11.61	13.55	24.84	6.52
1H-Indole	3.61	-	-	-	-
Jasmone	4.06	-	10.90	13.10	-
*Daehan Ujeon Wild*					
2-Ethyl-1-hexanol	-	2.32	-	-	-
Benzenemethanol	3.42	4.26	-	-	-
Linalool	-	3.87	-	-	-
Nonanal	4.06	4.97	6.19	-	6.77
1H-Indole	-	5.48	-	-	-
Jasmone	-	6.84	-	-	-
*Illohyang*					
(z)-4-Hexen-1-ol	2.84	1.87	-	-	-
Linalool oxide	4.06	3.29	-	-	-
Linalool	11.74	12.06	15.10	10.00	7.48
Nonanal	3.68	3.03	4.84	3.48	5.03
Geraniol	16.58	19.61	18.71	16.26	9.68
Jasmone	3.42	4.06	3.87	2.90	-
*Myoungjeon*					
4-Methyl-3-penten-2-one	1.42	-	-	-	-
Benzaldehyde	0.90	-	-	-	-
2-Ethyl-1-hexanol	-	-	-	-	4.52
Benzeneacetaldehyde	2.32	-	-	-	-
Nonanal	-	-	-	-	10.84
Jasmone	2.58	-	-	-	-
*Ouksu*					
4-Methyl-3-penten-2-one	2.65	2.45	-	-	-
Benzeneacetaldehyde	1.74	-	-	-	-
Linalool	-	-	3.16	-	-
Nonanal	1.74	3.74	4.45	-	7.81
Geraniol	1.23	-	-	-	-
Jasmone	3.35	2.52	-	3.68	-
β-Ionone	2.32	3.55	2.39	1.87	-
*Ujeon Okro*					
4-Methyl-3-penten-2-one	3.81				
Linalool	-	13.74	9.29	6.26	6.84
Nonanal	-	9.81	4.77	6.26	9.10
Benzeneethanol	-	4.39	-	-	-
Geraniol	-	7.23	4.58	3.10	-
Jasmone	-	5.48	3.55	2.58	-
β*-*Ionone	2.26	-	-	-	-

(*z*)-4-Hexen-1-ol was present in the *Illohyang* sample at the first and the second brews. However, no literature on green tea volatiles reported this compound and no information regarding the aroma or flavor characteristics was found. 

2-Ethyl-1-hexanol was detected in the *Daehan Ujeon Wild* sample at the second brew and in the *Myoungjeon* sample at the fifth brew only. The concentrations of the compound in these two samples were at 2.32 μg/L and 4.52 μg/L, respectively. It was previously reported in a Japanese green tea [15] but no other literature has reported 2-ethyl-1-hexanol in green teas. It has a mild, oily, sweet and slight rose fragrance [37]. 

Benzyl alcohol (benzenemethanol) was found in a few brews of the *Daehan Ujeon Wild Tea* sample and the *Daehan Ujeon* sample. It has a faint aromatic odor [37] and has previously been found in Korean tea [38]. Phenethyl alcohol (benzeneethanol) has a floral and rose odor [37] and was found in the second brew of the *Ujeon Okro* sample.

Linalool has been reported in green tea by many other researchers [15,38,39,40]. As expected, it was detected in all five samples except *Myoungjeon* and was present in more than three consecutive brews in the *Daehan Ujeon*, *Illohyang*, and *Ujeon Okro* samples*.* Though, the trend of changes in the concentration for repeated brews differed among these samples. Linalool’s odor is similar to bergamot oil [37] and citrus [41]. It is detectable at as low as 0.087 μg/L and recognizable at as low as 0.17 μg/L [41], which both are lower than our findings ranging from 3.16 μg/L to 15.10 μg/L. 

Geraniol is another terpene alcohol and has a geranium odor [37]. It was found in the *Illohyang* and the *Ujeon Okro* samples. The concentrations of geraniol in these two samples were the highest in the second brew and declined as they were brewed repeatedly. Geraniol has been found commonly in green teas [38]. Its thresholds were reported as 1.1 μg/L for detection and 2.5 μg/L for recognition [41].

Nonanal was present in all six samples in our study. However, the concentrations of nonanal in each brew did not have any perceivable trend. It has a tallow, fruity [42], strong, fatty odor [37], citrus-like and soapy [41]. Its detection threshold was reported as 2.8 μg/L and its recognition threshold was reported as 8.0 μg/L [41]. The nonanal was reported in a Korean commercial green tea made with tea leaves harvested in July and roast-processed [43] but was not reported in the other studies with Korean green teas [10,11]. 

Benzaldehyde was only detected in the first brew of the *Myoungjeon* sample. It is an aromatic aldehyde and has an almond odor [37] and commonly is used in artificial cherry flavor to provide a strong fruity note. The benzaldehyde was commonly found in green teas from Korea in previous literature [10,11,43]. The benzaldehyde may not be detected in more of our samples because of the sample preparation and the extraction technique. In the current study, brewed green tea liquor was used as a sample and the volatile compounds were extracted using SPME, which can detect the concentrations ranging from pg/g (ppt) to μg/g (ppm). The concentration of benzaldehyde in the second brew of the *Myoungjeon* sample was 0.90 μg/L , which is 0.90 ppb. 

Phenylacetaldehyde (benzeneacetaldehyde) was present in the first brews of the *Myoungjeon* and the *Ouksu* samples. It is an aromatic aldehyde and has a pungent, green, hyacinth-like, apricot, and berry-like flavor [37].

4-Methyl-3-pentene-2-one was detected in the *Myoungjeon*, *Ujeon Okro* and *Ouksu* samples. The concentrations ranged between 1.42 and 3.81 μg/L. It has a sweet, fruity odor and it is somewhat water soluble [37]. It was reported in the Korean green tea along with a *Chunmee* green tea and a *Gorreana* green tea [38]. 

Jasmone was found in all five samples except *Myoungjeon*. However, it is hard to find any trend in the concentrations. The jasmone was reported in the previous research [10,39]. The jasmone is an aliphatic ketone and has a jasmine odor and a fruity flavor [37]. 

β-Ionone was detected in four brews of the *Ouksu* sample but concentration was lower than detection threshold reported [41] except in the second brew. Others have found this compound in Korean green teas [11,39] as well as green teas from China [40] and Japan [15]. It has a woody odor [37], flowery and violet-like [41]. It was reported that one can detect the odor at 3.5 μg/L and can recognize it at 8.4 μg/L [41].

Linalool oxide was found in the first two brews of the *Illohyang* and the concentrations were around 4 μg/L. The linalool oxides were reported in the commercial Korean green teas made with tea leaves harvested in April and June [10] but not in August [11]. It has a sweet, lemon, cineol flavor [37]. 

1H-indole was detected in a few brews of the *Daehan Ujeon* and *Daehan Ujeon Wild* samples. The concentrations were all below 6 μg/L. It has a floral, animal, jasmine and earthy odor and it is volatile with steam [37]. The indole was also reported as having a fecal, mothball-like odor and the detection threshold was reported as 11 μg/L. In a previous study indole was reported as the most abundant volatile compound in the commercial Korean green tea samples, which were made with the tea leaves picked in April and June, but the concentrations were not reported [10].

### 3.3. Relationships between Descriptive and Aroma Volatiles Analyses in Green Tea Samples

**Figure 2:**
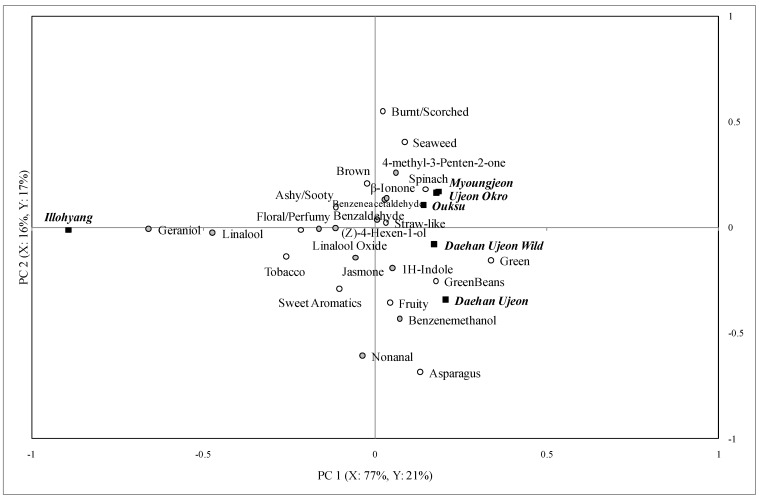
Partial least square regression analysis of descriptive sensory and gas chromatography data for the first brew of green tea samples.

**Figure 3:**
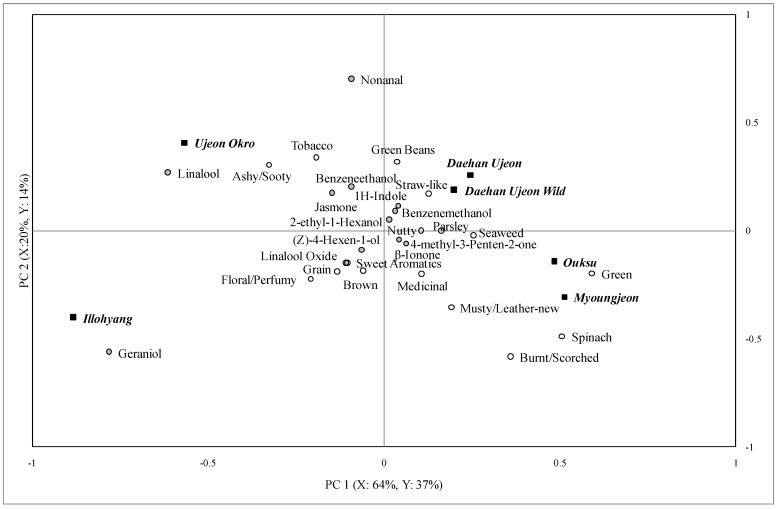
Partial least square regression analysis of descriptive sensory and gas chromatography data for the second brew of green tea samples.

The partial least square regression (PLSR) was conducted to relate the instrumental data to the descriptive data for each brew. The PLSR biplots of the first and the second brews are shown in Figure 2 and Figure 3. When terms on the map are closer they represent a higher relationship among the attributes and chemical composition. Of 25 descriptive attributes, only the aromatic attributes were included for the analysis (excluding bitter, astringent, and tooth-etch, which are not dependent on volatile compounds). Generally, the number of sensory attributes perceived, their intensities, and the number of volatile compounds detected decreased markedly from the second brew to the third brew.

Twelve volatile compounds were detected by GC-MS and 16 sensory attributes were perceived by the trained panelists in the first brew (Figure 2). The PLSR map shows that 95% of the instrumental data explained 38% of descriptive sensory data when the first two principal components were considered. The geraniol and linalool were explained mainly in the PC 1 and nonanal and benzenemethanol were explained in the PC 2. The geraniol, linalool and linalool oxide compounds were correlated with the floral/perfumy note. The nonanal, jasmone, and benzenemethanol compounds were related to the fruity and sweet aromatics notes.

Twelve volatile compounds and 16 sensory attributes were found in the second brews of the green tea samples (Figure 3). The PLSR results indicated that 84% of the instrumental data explained 51% of the descriptive sensory data in the first two PCs. Geraniol and linalool were the main vectors in PC 1 and PC 2 was explained by nonanal, geraniol, and linalool. Geraniol, nonanal, and linalool oxide were also related to the floral/perfumy attributes.

Only five volatile compounds, but 14 sensory terms, were used to describe the third brews of green tea samples. Around 96% of the instrumental data explained 25% of the descriptive data in the first two PC’s. It appears that the floral/perfumy attributes are related to the presence of geraniol, linalool, and jasmone but it is hard to know for sure because the explanation rate of the descriptive data by the instrumental data was only 25%. 

Five volatile compounds and nine sensory descriptive attributes were present in the samples brewed four times. The PLSR data showed that 88% of the instrumental data explained 37% of the descriptive sensory data. As expected, nonanal, linalool, jasmone, and geraniol were related to the floral/perfumy notes. 

Only four volatile compounds were detected using GC-MS and nine flavor attributes were perceived in the fifth brew of green tea samples. The PLSR analysis showed that 87% of the instrumental data explained 54% of the descriptive sensory data. In the PLSR biplot, it was hard to draw any relationship between the volatile compounds and the descriptive attributes probably because of the low levels of both the attributes and the aroma compounds that were found in the fifth brew.

## 4. Conclusions

The flavor changes of Korean green tea (leaf form) when brewed multiple times were measured using descriptive sensory analysis and GC-MS. According to our findings, the first and the second brews of green tea provided similar strengths of flavor intensities and the third and the fourth brews provided mild flavor, low bitterness, and low astringency. The fifth brew had a few flavor notes at low intensities, low bitterness, and low astringency, suggesting that the primary green tea flavors were gone by the fifth brew. Thus, based on this initial study, four brews would seem to be the maximum number of brews that can be recommended. In the brewed liquor of green tea mostly linalool, nonanal, geraniol, jasmone, and β-ionone volatile compounds were present at low levels. The geraniol, linalool, and linalool oxide compounds in green tea may contribute the floral/perfumy flavor. This result is true for high quality green teas produced in South Korea. Further research is needed to confirm our findings using different qualities of green tea from other sources and grades.
